# Separateness or wholeness: a qualitative study of the experiences of Asian American female sexual minorities

**DOI:** 10.3389/fpsyg.2024.1440858

**Published:** 2024-08-30

**Authors:** Bin Zhang, Gilbert Park, Bo Chang, Wenqian Du

**Affiliations:** ^1^School of International Education, Shandong University, Jinan, China; ^2^Teachers College, Ball State University, Muncie, IN, United States

**Keywords:** race, gender, sexuality, intersectionality, objectification, Asian American, qualitative study

## Abstract

This study explored the intersection of race, gender, and sexuality as they pertain to experiences of Asian American female sexual minority (AAFSM) students attending Midwestern universities in the United States through an intersectional lens. The study utilized intersectionality as a theoretical framework, a data generation tool, and a methodological approach to guide the study. The results showed that the participants experienced constructed objectifications, which included gendered, racial, and sexual objectification. The findings also revealed that participants’ race, gender, and sexual orientation were contextualized based on the situation. Further, participants devalued their Asianness, womanhood, and LGBTQness owing to the lack of positive representation in the curriculum. The analyzed data can be best categorized as the lack of intersectional representations in curricula, contextualized race, gender, and sexuality, and reported experiences of constructed objectifications. Discussions provided an inclusive campus environment for participants who were AAFSMs. These discussions also provided meaningful suggestions for educators, administrators, policymakers, and stakeholders to foster an equal and equitable educational environment for students with multiple marginalized identities.

## 1 Introduction

After surveying the literature on AAFSM college students’ experiences, it became apparent that few studies had explored their experiences in the predominantly White Midwestern universities in the United States. A review of extant literature demonstrated the inadequate amount of research on this population. Recent scholarship had primarily presented any two marginalized categories (out of race, gender, sexual orientation, social class, and other types of social subordination) as closely interwoven and intertwined. Analysis of the lived and educational experiences of AAFSM college students had been overlooked, as indicated by the insufficient amount of research. Additional scholarly work exploring AAFSM college students’ lived and educational experiences can contribute to the growing body of the existing literature. Additionally, it will serve to raise awareness among higher education policymakers of the complex issues involved with intersectionality, and to provide points of intervention to meet the particularities of the perspectives and needs of AAFSM students.

Therefore, this study aimed to draw attention to an unmarked marginalized group—Asian American female sexual minorities (AAFSMs)—and to explore their lived and educational experiences in Midwestern universities in the United States. Particularly, the study explored how AAFSM’s personal identity, college experiences, and educational opportunities were co-constructed with race, gender, and sexual orientation.

When examining multiple marginalized subjects within feminist theory and anti-racist policy discourse scholarship, Crenshaw (1989) contented that single-axis analysis (e.g., focusing solely on gender or race) did not suffice. Crenshaw critiqued previously published material for merely utilizing a single analytical category to understand African American women. For example, feminist theory commonly emphasized White women, and anti-racist policy discourses tended to emphasize racial discrimination from the perspective of men of color.

Additionally, Crenshaw asserted that merely adding one subordinate category onto another (e.g., African American plus women does not equal to Black women) does not authentically reflect Black women’s lived experiences. Black women’s lived experiences demonstrate that “the intersectional experience is greater than the sum of racism and sexism, any analysis that does not take intersectionality into account cannot sufficiently address the particular manner in which Black women are subordinated” (Crenshaw, 1989, p. 140). Crenshaw’s criticism and arguments regarding the inequality of Black women lead to the key insight of intersectionality, which calls for researchers to remove additive thinking when utilizingintersectionality.

During the late 1980s and early 1990s, scholars such as Crenshaw (1989) reconsidered inequality and realized that it should not be understood solely along one dimension, such as race or gender. A noticeable absence of intersectionality-related discussion on inequality existed during this period. Ferree (2018) argued that a foundational sociological understanding of inequality did not account for the intersection of race, gender, and class. However, Crenshaw (1989) highlighted an important exception when employing the concept of intersectionality to examine inequality as it pertains to the lived experiences of African American women, establishing them as prototypical intersectional subjects for the subsequent decades.

Crenshaw (1989) critically advanced that intersectionality, as a transitional concept and methodology, should remedy how mutually exclusive categories are falsely separated to counter the disembodiment of multiple oppressed groups at the intersection. Since then, the term “intersectionality” has been utilized by scholars across various venues and has become a “buzzword” (Davis, 2008, p. 75; Nash, 2020, p. 120). However, within academia, intersectionality should not be utilized as a “buzzword” or over-hyped trend. Instead, intersectionality is intended to challenge the prevailing mindsets of the dominant groups and to disrupt the normative claims of dominant discourses (e.g., heteronormativity, masculinity, and Whiteness). Intersectionality should “disorient” us to “get us thinking about how ‘we’ think” (Carastathis, 2016, p. 111). Drawing on Ahmed’s (2006) concept of disorientation, intersectionality should be deployed in research to deconstruct and disorient our entrenched cognitive habits. For instance, how does one understand and make sense of normalized and exalted identities (e.g., male, heterosexuality, White, etc.) compared with pathologized and repressed identities [e.g., female, lesbian, gay, bisexual, transgender, and queer and/or questioning (LGBTQ), non-White]?

In the subsequent sections of this article, we employed intersectionality as our theoretical lens to explore the nuanced experiences of AAFSM students. We reviewed literature focusing on racism, heterosexism, and sexism in relation to AAFSM students to establish a foundational understanding of the pervasive issues of racism, heterosexism, and sexism, and to highlight the gaps and challenges that our study seeks to address. We described our research methodology, detailing the process of participant selection, data collection, and analysis that shaped our study. We presented findings categorized into themes, reflecting the core issues faced by AAFSM students. We discussed the findings and concluded with the main ideas generalized from this project. Finally, in implications, we provided recommendations for educational practices and policies on inclusivity and equity and pointed out the contributions of our study to the theory and practice.

## 2 Theoretical framework

This study employed the intersectionality theoretical framework, drawing from a brief discussion of the limitation of each of above-mentioned theories.

Feminist theory has been utilized in studies exploring various facets by feminist researchers, encompassing women’s voices (Leavy, 2020), women’s empowerment and social change (Letherby, 2003), girls’ middle school experiences (Finders, 1997), and gender studies (Jaggar, 2015). Furthermore, feminist theory is broadly associated with “specific disciplines and with the writings of women of color; intersectional feminism, including the intersection of racism, sexism, homophobia, trans-phobia, ableism, xenophobia, and classism; women problematizing Whiteness; postcolonial, transnational discourse; decolonizing arguments of indigenous women; lesbian, gay, bisexual, transgender, and queer and/or questioning (LGBTQ); disabled women…” (Denzin and Lincoln, 2018, p. 99). Although feminist scholars have contributed to the development of a more inclusive academic world through feminist transformative developments, the primary focus addressed by feminist scholarship is typically that of women (DeVault, 2018). Notwithstanding, feminist scholarship has begun to incorporate other marginalized identities besides women, though many scholars still raise questions about whether (White) feminist scholarship can authentically capture and comprehend women’s marginalization (Cole, 1986; Hooks, 2000; Woo, 1985). With this said, feminist theory is insufficient to explore the experiences of women who stand at the intersection of race, gender, sexuality, religion, nationality, disability, and other factors. As Parker and Lynn (2002) articulated, “In the case of Black women, race does not exist outside of gender and gender does not exist outside of race” (p. 12). This leads to the second discussion regarding the limitation of critical race theory in its application to the study.

Critical race theory specifically focuses on race, racism, and power dynamics, along with their relationships and transformation among them (Delgado, 2017). Race and racism constitute normal components of American life and are deeply embedded in American society (Ladson-Billings, 2003; Parker and Lynn, 2002). The literature unequivocally highlights one thing: students of color have faced discrimination, harassment, macro-and micro-aggression based on race at both individual and institutional levels for decades (Alvarez, 2009; Brunsma et al., 2017). Consequently, critical race theory has been widely utilized to examine campus climate (Solorzano and Yosso, 2001) and experiences of Black students (Duncan, 2002) in the hopes of dismantling oppression and fostering inclusivity. However, the inclusion that is advocated by critical race theorists is arguably called limited or imperfect inclusion by the post-racial theorists (Kim, 2015). For instance, the inclusivity benefiting men of color might inadvertently exclude women of color, among others. Critical race theory may be utilized to emancipate Asian American men but may oppress Asian American women who are also members of LGBTQ groups. Therefore, limitations of critical race theory in exploring the experiences of AAFSM stand out significantly.

Queer theory, in contrast to gay, lesbian, or homosexual theory, endeavors to keep all sexual categories inclusive (Creswell and Poth, 2016; Leavy, 2020). These sexual categories include, but are not limited to, gay, lesbian, bisexual, questioning, and others. The term queer itself specifically refers to being “outside the norms” (Leavy, 2020, p. 93). However, the terms lesbian and gay have been constructed based on norms that enforce a binary gender, which is inadequate to describe women who are sexual minorities. For instance, some women with distinct masculine traits identified themselves as gay instead of lesbian. Other examples abound: some women self-identified as lesbian while acknowledging their biological gender, while some did not. As Creswell and Poth (2016) argued, “The historical binary distinctions are inadequate to describe sexual identity” (p. 31). Therefore, this study chose to use the term AAFSM instead of Asian American lesbian because the former identity is more open and less constrained. Similar to critical race theory and feminist theory, queer theory is inadequate to explore Asian American female sexual minorities.

According to the preceding discussions regarding multiple theoretical frameworks, the intersectionality framework was employed to design the study, frame research interview questions, and guide data generation and analysis. Collins and Bilge (2021, p. 25) delineated, “Intersectionality is a way of understanding and analyzing the complexity in the world, in people, and in human experience.” Intersectionality provides the most appropriate theoretical framework to shed light on the complexity of educational experiences of AAFSM college students at the intersection of race, gender, and sexual orientation. It has been extensively employed to articulate the intersecting oppression, complex relationships of power and oppression, and social locations shaped by analytical identity categories such as race, gender, and sexual orientation. There are three reasons for its extensive employment. First, it stems from the nature and attributes of analytical identity categories, which intersect. The second reason lies in the social systems and power, which intersect, constitute, and reconstitute each other. The third reason is the nature of the intersection of interlocking systems and intersecting analytical identity categories. In line with the purpose of applying the intersectionality framework in this study, it is crucial to review the core ideas of the intersectional framework. These core ideas include, but are not limited to, social inequalities (i.e., racism, sexism, and hetero-sexism), power (i.e., heteronormativity, White supremacy, and masculinity), and relationality (Collins and Bilge, 2021; Windsong, 2016).

## 3 Literature review

### 3.1 Racism

The United States society has an invisible but powerful pyramidal hierarchy; situating people of color at the bottom while endowing and privileged White/European Americans at the top. This pyramidal hierarchy, rooted in race, serves as a pervasive social force in the United States, socially referred to as racism. To facilitate readers’ comprehension of racism in general and anti-Asian American discourses in particular, it may be more beneficial to examine racism within the historical context of the United States. During the late 19th century, Asian American immigrants were recruited as cheap laborers in plantations of Hawaii, the canneries of Alaska, and the mines of California (Alvarez, 2009). Despite being recruited by American plantations, Asian American immigrants were constantly viewed as “protentional threats to national security” because they were perceived as the “yellow peril,” who would supposedly take jobs away from real Americans (Lee, 2005, p. 5; Lowe, 1996). Therefore, anti-Chinese movements and discourses intensified (Wollenberg, 2014). Early Asian immigrants, particularly Chinese immigrants, were portrayed as “nothing more than starving masses, beasts of burden, depraved heathens and opium addicts” (Chan, 1991, p. 45).

Jones (1997) categorized racism into three facets: individual, institutional, and cultural racism. Specifically, individual racism refers to a social dynamic that operates with a superior racial group working against a racial group deemed inferior. It includes, but is not limited to, acts of bullying, verbal and physical harassment, and discrimination. In other words, racial minorities may experience anything from overt verbal and physical discrimination at its worst and microaggressions at its mildest. For instance, recent studies by Ong et al. (2013) have demonstrated that Asian American college-aged students have experienced overt racial harassment due to their race or ethnicity. In a recent study, 32 percent of Asian Americans reported being the target of racial slurs, and over one third reported experiencing microaggressions based on race (McMurtry et al., 2019). Specifically, Asian Americans experienced microaggressions in the form of discriminatory slurs, such as yellow peril, model minority (Lee and Hong, 2020), forever/perpetual foreigners, honorary Whites, (Tuan, 1998), and Otherness/Others (Wooden et al., 1983). These racial slurs, as components of an anti-Asian American discourse, denied Asian Americans the ability to identify themselves as pure Americans, regardless of the number of generations their families have been in the United States (Lee, 2005).

Although Asian Americans were often portrayed as forever/perpetual foreigners, White/European Americans sometimes regarded them as “honorary Whites” (Tuan, 1998), aligning them with White. Nevertheless, Asian Americans have never been fully accepted as authentic Americans. Therefore, it is crucial to recognize that Asian Americans, despite being labeled as “honorary Whites,” do not enjoy the same privileges as White Americans (Lee, 2005). The racial slur “honorary Whites” was extended from another race-based slur—moral minorities—which has long been associated with Asian Americans. Since the 1960s, Asian Americans have been subjected to racial prejudices and model minority stereotypes owing to their reputation for hard-worker and high academic achievements, such as being perceived as “good at mathematics” and academically inclined (Qin et al., 2008; Yeo et al., 2019). While some Asian Americans may view these stereotypes as positive compliments, the majority feel insulted and pressured by being unfairly labeled in this way (Yeo et al., 2019). For instance, in a study, an Asian American male student named Feng explained the insult:

People usually ask me for help in mathematics or sciences. Then if I get their question wrong, they get mad at me and say, “Aren’t you supposed to be good?” I felt like I was a disappointment to this premade stereotype they had of me (p. 55).

Therefore, the racialized stereotype (i.e., model minority) serves to oppress and suppress Asian Americans and keep them marginalized, under-served, and unrepresented.

In addition to individual racism, Asian Americans were also targeted by institutional racism. Institutional racism in the form of policies—consciously and subconsciously, intentionally and unintentionally, and covertly and overtly—affects racial minorities. For instance, historically, second generation Asian American immigrants were prohibited from attending San Francisco public schools during the late 19th century by the San Francisco Board of Education policy (Wollenberg, 2014). Since the 1990s, some prestigious universities, including, but not limited to, Harvard University, Stanford University, UC Berkeley, and Princeton University, have been accused of racism owing to their differential acceptance of Asian American students (Alvarez, 2009). Recently, McMurtry et al. (2019) indicated that approximately one in six Asian adults reported “experiencing discrimination when applying to or while attending college…” Other examples abound, such as Kim, a female Asian American student from the study by Yeo et al. (2019), indicated that a majority of people on campus held a negative connotation toward Asian Americans. The negative connotation, which was further elaborated by another interviewee in the study, included “There are too many Asians on campus. We do not need more” (p. 50). The above-mentioned instances demonstrate that institutional barriers against Asian American students can be found in the educational system in many aspects. In short, both the historical and contemporary evidence indicate that institutional racism exists in the form of policies, regulations, and laws constantly affecting racial minorities.

Parallel to individual and institutional racism, Asian Americans, for instance, experience cultural racism implicitly and explicitly. One type of cultural racism manifests in how peers perceive “good” and “bad” students in American schools. Lee (2005) delineated, “Traditional and Americanized [Hmong] youth defined themselves against each other” (p. 53). Traditional Hmong students were advised to stay away from “bad” Hmong kids who were too Americanized, while the Americanized Hmong youths viewed Hmong students maintaining traditions as problematic in the United States. This within-group conflict stemmed from two cultures. One was the home country culture of Hmong students, which was considered problematic; the other was American culture, which was valued as cultural norms and promoted the dominance of Whiteness over other races. In effect, these dominant beliefs and values enhanced White dominance, (re)producing and fueling racism; in turn, racism reinforced these dominant beliefs and values, empowering Whiteness but disempowering other races.

To summarize, this section provides an overview of Asian Americans’ experiences with racism from three facets: individual, institutional, and cultural racism. Some instances from the literature illustrate how these forms of racism affect Asian Americans’ lives. The discussion also explains that racism both shapes and is shaped by other marginalized identities, such as gender. Merely focusing on race to research racial minorities’ experiences, identities, and other unexplored fields is not sufficient. Thus, employing intersectionality to explore AAFSM college-aged students’ educational experiences is necessary to contribute to the body of literature.

### 3.2 Heterosexism

In addition to racism, heterosexism is another social force that needs to be addressed. The majority of studies have revealed how deeply heteronormativity is ingrained in United States educational institutions including both K-12 and higher education. Heterosexuality is viewed as natural and desirable, whereas homosexuality is perceived as otherness (Kitzinger, 2005; Woody, 2003). Consequently, heterosexism normalized heterosexual behaviors, activities, beliefs, and values while demonizing, denigrating, and rejecting homosexual forms (Herek, 1995). Due to this social force and oppression, many students in the U.S. experienced discrimination, oppression, harassment, and exclusion to varying degrees. Similar to racism, heterosexism “may be expressed overtly or covertly, consciously or unconsciously, and intentionally or unintentionally” (Miville and Ferguson, 2006). In other words, instances of heterosexism can range from unintentional, unconscious, or covert forms (e.g., “I like your jeans—no homo”) to intentional, conscious, and overt forms (e.g., being punched, kicked, injured with a weapon, or threatened by a bomb) (Kosciw and Cullen, 2002; Kosciw, 2004; Kosciw and Diaz, 2006; Kosciw et al., 2008, 2010, 2012, 2014, 2016, 2018). These instances, in turn, (re)produce and reinforce the ideology and notion that heteronormativity is the social norm, superior, and privileged, while those who do not conform to heterosexual beliefs, values, and standards are deviant, abnormal, and sick.

Why is addressing issues of heteronormativity and heterosexism such an important topic in education? LGBTQ youth and college-aged students frequently report experiencing discrimination, harassment, exclusion, and violence in schools, colleges, and universities. These negative experiences affect students in various ways to different degrees. To provide readers with a deeper and holistic understanding on how heterosexism impacts students, findings from 10 different reports are summarized (see Table 1 and also the references of these reports). According to these 10 reports, the challenges that LGBTQ students face in schools, colleges and universities are numerous and diverse. From a national perspective, both LGBTQ students in school and college campus experienced verbal and physical harassment, overtly and covertly, at significantly higher levels than their heterosexual counterparts. Additionally, LGBTQ students, both young and college-aged, are frequently targeted by homophobic and similar derogatory remarks. Specifically, according to the National School Climate Survey reports on LGBTQ youths’ school experiences in United States schools in 2002, 2004, 2006, 2008, 2010, 2012, 2014, 2016, and 2018, LGBTQ students experienced verbal and physical harassment based on their sexual orientation in the past school year (please check the percentages of verbal and physical harassment for every other year from 2001 to 2017 in Table 1). The data clearly illustrate that homophobic harassment and victimization experiences have become a common experience for many sexual minority students in K-12 schools.

**Table 1:** Percentage of Lesbian, Gay, Bisexual, Transgender or Questioning/Queer (L, G, B, T, Q) respondents reporting particular experiences related to campus climate in schools and university campus.

Academic year		K-12									College			
Experiences reported		2000–2001%	2002–2003%	2004–2005%	2006–2007%	2008–2009%	2010–2011%	2012–2013%	2014–2015%	2016–2017%	2000–2001%, (*n*)	2009–2010%		
		L, G, B, T							L, G, B, T, Q		L, G, B, T	L, G, B, Q	T	
													Transmasculine	Transfeminine
Felt unsafe/uncomfortable at schools due to their sexual orientation identity		68.6	64.3	64.3	60.8	61.1	63.5	55.5	57.6	59.5	19.0 (313)	70.0	56.0	n/a
Experiences of verbal harassment		83.3	84.1	83.1	86.2	84.6	81.9	74.1	70.8	70.1	48.0 (224)	23.0	39.0	38.0
Experiences of physical harassment		41.9	39.1	37.8	44.1	40.1	44.7	36.2	27.5	36.7	11.0 (53)			
Experiences of physical assault		21.1	16.9	17.6	22.1	18.8	18.3	16.5	13.0	12.4	2.0 (11)	n/a		
Experiences of hearing homophobic remarks at schools from students		99.2	99.9	98.8	99.6	99.7	99.6	99.0	99.0	91.8	74.0	61.0	n/a	
Experiences of hearing homophobic remarks at schools from faculty (i.e., teachers, staff, etc.)		62.8	60.6	56.0	63.0	60.4	56.9	51.4	56.2	56.6		n/a		
Avoided disclosing their sexual orientation due to a fear of negative consequences, harassment, or discrimination		n/a									34.0 (574)			
Location of harassment	Public space on campus	n/a									63.0			
	Their place of residence	n/a									40.0			
	Classroom	n/a									30.0			

This table summarizes conclusions from Kosciw and Cullen (2002), Kosciw (2004), Kosciw and Diaz (2006), Kosciw et al. (2008), Kosciw et al. (2010), Kosciw et al. (2012), Kosciw et al. (2014), Kosciw et al. (2016), Kosciw et al. (2018), Rankin (2003), and Rankin et al. (2010).

In comparison with data on K-12 school climate toward LGBTQ students, the comprehensive data on campus climate toward LGBTQ college students are limited. As demonstrated in Table 1, national studies on K-12 school climate toward LGBTQ youth and their experiences have been available for over a decade, but comprehensive information and data on higher education were lacking before 2010 despite the documentation of the hostile environment that LGBTQ college students often experience in numerous studies since the mid-1980s (Rankin, 2003; Renn, 2015). Rankin et al. (2010) indicated that, prior to 2010, many campus climate studies were conducted; however, most focused on a single institution, a small number of campuses, or a small group of LGBTQ people. The 2010 publication of the National College Climate Survey (Rankin et al., 2010), the most comprehensive national research study of its kind thus far, bridged the gap by extending the research from a single institution study to a broader national picture (Renn, 2015). However, how can K-12 and higher education LGBTQ research be connected? This question has also been posed by many researchers, such as Renn (2015). As presented in Table 1, the percentage of LGBTQ college students experiencing both verbal and physical harassment was lower than K-12 students in schools. Although this comparison may be problematic since participants from National College Climate Survey (Rankin et al., 2010) differ from those in the National School Climate Surveys (Kosciw and Cullen, 2002; Kosciw, 2004; Kosciw and Diaz, 2006; Kosciw et al., 2008, 2010, 2012, 2014, 2016, 2018), it still provides evidence that college students reported significantly fewer incidents than K-12 students did. The main reason for this difference is that many LGBTQ college students were reluctant to disclose their sexual orientation due to the campus environment that can cause anxiety and depression. The more they opened up about their sexual orientation, the more likely they were to experience harassment, discrimination, and assaults. As Yeskel (1985) indicated, “The lack of adequate physical protection, the anti-gay stance of many academic courses, the inadequacy of student services and the openly anti-gay atmosphere in many residence halls combine to create a climate producing anxiety and depression for many of these [LGBTQ] students” (p. 21). Therefore, data on college students with a sexual minority identity were limited. Exploring the experiences of college students with a sexual minority identity is imperative to answer such questions.

### 3.3 Sexism

Sexism, being a social oppressor, is closely connected to heterosexism in that it further perpetuates the binary gender form. The conformity of this binary gender form creates an oppressive gender hierarchy, dominated by White males, and positions women as experiencing coercion, subordination, and submission. For instance, according to Miville and Ferguson (2006), much research has indicated that the attributes ascribed to men were active, rational, and inventive; whereas women were weak and gentle. To be sure, sexism and sexist stereotypes appear to be fairly common across most racial and cultural groups. In other words, White women, women of color, poor women, and the like, experience various manifestations of sexism ranging from covert to overt, conscious to subconscious, or intentional to unintentional. However, there exists a tremendous disparity with regard to sexism between White women and other women (i.e., women of color). It is also important to remember that experiences of women of color worsen when their racial identities are taken into consideration.

Specifically, Asian American women need to address conflicts between traditional and nontraditional cultures. Traditionally, Asian culture measures a woman’s value based on three tenets of obedience—“obedience to father, submission to the husband, and indulgence of the son” (Hall, 2009, p. 196). Fully complying with these three obedience standards was deemed the way to become an Asian American woman regarded as a good daughter, wife, and mother. Simultaneously, Asian American women were required to join in the wave of the women’s liberation movements in the US to portray themselves as individual and independent. Therefore, as Hall (2009) stated, “[B]alancing the traditional with the nontraditional has become a major stress factor for Asian American women” (p. 196). Similar to Hmong youths, who had to balance their traditional and American culture to survive in US schools, Asian American women had to navigate between their racial culture in terms of gender expectations and American culture regarding gender as well. Both cultures could incur sexisms toward Asian American women. The manifestations of sexism are different: one is racialized sexism; the other is gendered sexism. Although this section discussed sexism, sexism does not solely revolve around gendered activities, but is also influenced by other factors.

## 4 Research methodology

Phenomenology has been selected as the methodological approach for this research project to delve deeply into the shared lived experiences of individuals who navigate multiple marginalized identities. Phenomenology is particularly suited for this inquiry because it focuses on uncovering the common essence of experiences, allowing for a nuanced understanding of how intersectional experiences manifest across different individuals (Creswell and Poth, 2016). Phenomenology seeks to distill the essence from these personal accounts, thereby illuminating the pervasive nature of intersectionality based on race, gender, and sexual orientation (Crenshaw, 1989; Han, 2017; Robinson, 1999). This aligns with the objective to produce a composite description that captures the collective experience of intersectional identities. Additionally, phenomenology’s emphasis on reducing individual experiences to an essence ensures that the findings transcend personal anecdotes, providing a robust and objective scientific analysis of these subjective experiences (Spencer et al., 2015). Thus, phenomenology enables a comprehensive exploration of the phenomenon, translating subjective lived experiences into an “object” of human experience (Merriam and Tisdell, 2016; Creswell and Poth, 2016), making it the most appropriate methodology for this study.

### 4.1 Research participants description

This study aimed to explore the lived and educational experiences of AAFSM college students in Midwestern universities in the United States. To achieve this objective, specific participation criteria were used to identify individuals within the target population. The criteria for participant selection were as follows, to purposively sample the population:

1. Individuals possessing multiple marginalized identities—Asian American, female, and sexual minority.
2. Individuals demonstrating a strong interest in participating in the study.
3. Individuals currently enrolled in predominantly White Midwestern universities.

Participant recruitment employed snowball sampling and a participant-to-researcher method. Participant recruitment employed snowball sampling and a participant-to-researcher approach. The research proposal was presented at multiple local, regional, and state conferences to facilitate recruiting participation. Following a presentation at a local conference, the initial participant expressed interest in joining the study. Additionally, informed consent forms were distributed through diverse channels, including local student organizations, social media groups, and networks supporting Asian, women, and LGBTQ+ students. As a result, nine AAFSM students from Midwestern universities in the United States were successfully recruited.

All participants were asked to self-identify their race, gender, and sexual orientation through a confidential survey. The survey included options to ensure inclusivity and respect for all identities, such as: Asian, non-white, American, white, female, male, non-binary, heterosexual, homosexual, bisexual, pansexual, asexual, queer, other, and prefer not to say. Participants could select the option that best described their identity or provide their own term if it was not listed. This self-identification approach allowed participants to express their identities accurately and comfortably.

The participants’ details are listed in Table 2.

**Table 2:** Participants’ demographics.

Categories	Race	Gender	Sexuality	Population
Self-identified as	Chinese American	Female	Bisexual	3
	Chinese American	Fluid	Demi-homosexual	1
	Filipino American	Female	Bisexual	2
	Filipino American	Female	Queer	1
	Asian American	Fluid	Bisexual	1
	Korean American	Non-binary	Lesbian	1

### 4.2 Data collection

This study utilized qualitative data collection methods and employed the Dynamicity and Complexity (D&C) model (Zhang et al., 2021) as the interview method. The D&C model integrates various philosophical approaches and interview structures to enhance intersectionality research. Drawing from Roulston (2010) and Alvesson and Deetz (2000), the model incorporates different epistemological positions—neopositivist, romanticist, localist, constructionist, and postmodernist—into the interview process. Each approach offers distinct ways to explore intersectional issues. Interviews can be structured, semi-structured, or unstructured. Structured interviews use a rigid format, potentially limiting depth, while unstructured interviews allow for more detailed, personal insights. Semi-structured interviews provide a balance, guiding the conversation while allowing flexibility. In this study, the D&C model was applied dynamically, switching between these approaches and structures as needed.

Data were collected through one-on-one interviews with AAFSM college students, tailored to suit the research context and ensure comprehensive and effective data collection on complex intersectional issues. Each interview lasted from 1 to 2 h, during which participants responded to open-ended questions to provide narrative data. Structured, semi-structured, and unstructured follow-up questions were employed to delve deeper into responses. Interviews continued until information saturation was achieved. At least one follow-up interview, either by phone or face-to-face, was arranged for clarification and further explanation. All interviews were meticulously audio-recorded and transcribed to generate data, which was then coded, clustered into themes, and summarized.

### 4.3 Data analysis

The data analysis encompassed several steps, including data organization, an initial database review, theme coding, data representation, and interpretation formulation. Throughout this process, the qualitative data analysis software MAXQDA 2020 was employed to aid in the analysis. Within MAXQDA 2020, data were analyzed using Saldaña’s (2021) coding methods, which included process coding, narrative coding, dramaturgical coding, causation coding, value coding, domain and taxonomic coding, emotion coding, versus coding, concept coding, initial coding, *in vivo* coding, descriptive coding, subcoding, magnitude coding, and attribute coding. Utilizing these coding methods, 1,089 code segments were generated during the first coding cycle. In the second coding cycle, themes began to emerge.

### 4.4 Trustworthiness

To interpret the data accurately and ensure the trustworthiness of analysis, triangulation needs to be carefully considered. Denzin’s idea regarding of what he called “investigator triangulation,” which employs different people to control or correct subjective bias, is endorsed in this study (Denzin and Lincoln, 2018). To ensure the trustworthiness of the study, member checking was employed. After analyzing the data and concluding the findings, I asked five randomly selected participants out of the nine interviewed to review the themes, findings, and conclusions. This process was implemented to verify the accuracy and address any potential biases in the interpretations. All five participants confirmed that the findings accurately reflected their expressed experiences, affirming the validity and reliability of the study’s conclusions. For instance, member checking by asking participants in the research to review the themes, findings, and conclusions were used to verify the interpretations for accuracy and lack of bias. In addition to research participants, a legal scholar also reviewed the themes to address any potential subjective bias. In addition to investigator triangulation, a second type of triangulation is introduced by Denzin: methodological triangulation. This approach requires reviewing the data several times, each time applying different methodological lens to analyze data. For instance, the phenomenological methodological approach can be used to check the essence. Lastly, this multi-cases-study has data triangulation embedded in it. This study collected data in multiple places at different times with different individuals. During the data analysis process, several cases were examined independently to ensure the trustworthiness of the study through data triangulation.

## 5 Findings

### 5.1 Theme 1: lack of intersectional representations in curricula

As mentioned in the previous section regarding institutional racism, sexism, and heterosexism through curricula, the predominant White culture and/or Eurocentric character in the curriculum (re)produce and reinforce inequalities among students, leading to a “clash of cultures.” The student body comprises not only White heterosexual male students but also racial, gender, and sexual minorities. Therefore, curriculum should enculturate marginalized students into values required by mainstream cultures (i.e., White, heteronormativity, and patriarchy). Instead, it should be changed to better serve marginalized students by increasing the representations of people with intersectional identities. Institutionally, the lack of intersectional representations in curricula has played a role in excluding and blinding this study’s participants on campus. For instance, one participant, a Korean American female lesbian shared,

But then as I started doing more research and started thinking about okay… I never saw myself in the curriculum. I never… we never read books about Asian American lesbian. We never um you know talked about my culture, my history, or what it even is like to be an adopted person (Dale, Pos. 87).

Other participants agreed that the lack of intersectional representations in curricula on campus does not bring race, gender, and sexuality to “come to people’s mind” (Merritt, Pos. 64 and Arrow, Pos. 120).

Many participants also shared their concerns about speaking out to make themselves visible rather than remaining unseen. Participants admitted that they were hesitant to speak out because there were few representations in curricula. One participant indicated,

When it comes to like education side of things, um when it comes to like representation, like they have lots of people to do the represent representation to speak out. And a lot of them aren’t afraid to do that because like they’ve been doing it for years. They [African Americans] have representations in curricula like Martin Luther King. But as an Asian American bi woman, I can’t speak out. There were no representations in curricula to like to be my role model… (Charlie, Pos. 250).

Another participant from a different predominantly White Midwestern university further added that people did not really pay attention to them because they had no representation.

And there’s just not really have representation in curricula. Um So that’s … that’s the one thing um I mean like. There’s the whole thing of. Um yeah. Not really paying attention to Asian American lesbian students because we’re not seeing in curricula … we’re not seeing everywhere … (Lyric, Pos. 148).

This study concluded that the lack of intersectional representations in curricula facilitates the silencing of women’s voices, blinds people of color, and denies LGBTQ individuals. This lack of intersectional representation in curricula also contributes to intersectional blindness because “people did not really pay attention to them” (Lyric, Pos. 148).

### 5.2 Theme 2: contextualized race, gender, and sexual orientation

Bell hooks, a proponent of intersectional study, however, admitted that it was realistic for one identity to be foregrounded by an individual. The reason why individuals foregrounded their one identity over another could be because they were either forced or inadvertently asked to choose. Miville and Ferguson (2004) contended that sexual minority people of color are often forced to choose one community over another, such as religion, LGBTQ community, or their racial community. In this regard, people of color negatively developed a sense of separateness rather than a positive sense of wholeness.

In this study, all participants reported that identity rankings were not actively practiced or performed by individuals themselves; it was forced by privileged people. The identity ranking was subconsciously discussed based on the context, situation, and the people involved in the conversation. The fact was that race, gender, and sexual orientation could not be ranked, according to participants’ responses. The evidence was captured and showed as follows,

Oh, no. there is no way to rank them cause they’re all just like a part of me. And it’s not like something I could rank as in like, like my hair versus my eyes versus like, I don’t know, like things that I can like I could put in contacts, change my eyes. I can like color my hair to change my hair. But I can’t change my race. I can’t just take it off at the end of the day, I can’t change how I feel about people that I love romantically or sexually. I cannot change that. I had those feelings. And I can’t change that. I and the gender I that I am not confirmative to a specific gender. And if like I need to have that freedom of being the gender and expressing how I am, or else I feel like I am restricted. I cannot take any of that off at the end of the day. It is a part of me forever. And it is an important part of me as a human (Charlie, Pos. 94).

Nevertheless, many participants admitted that they foreground either race, gender, or sexual orientation on many occasions. They also admitted that they had to oppress some part of themselves due to the context and situation. Both foreground and background identities produced micro-and macro-oppression toward the study participants, who were AAFSMs. Visible identity produced visible exclusions, while invisible identities caused invisible exclusions. These intersectional exclusions made people invisible in many communities.

When the authors asked another participant, who was interviewed, if her of identities was placed at foreground, she elaborated,

I think that’s a really interesting question because I do actually rank them depending on uh like we were talking about earlier context matters. So, say, for example, I’m in America and I walk into a room and it’s only white people then definitely being Asian and my race is at the foreground. Whereas if I walk into a room and there are several Asian people and a couple other races and what not… then being an Asian and a woman kind of rank on the same scale. And like uh sexual orientation is kind of in the background a little bit just because it’s not as visible. And then um if we’re in a room full of Asian people or Filipino people, then being a woman is the foreground. And then I think in all of these situations, sexual orientation tends to take the background just because it’s more invisible. Like it’s not very obvious that I’m in that community. So um it tends to take the backseat unless we are actively talking about it. Um say, for example, a class session is about the LGBTQ community. Then I think that identity is on equal weight as the other ones. But I think in America, in almost all contexts, being Asian is the most salient. And then being a woman comes in a close second and sometimes equal depending on the context. Um but I definitely had situations where being a woman is like on the foreground just because someone’s being sexist. So, I’m like okay time to leave. But it depends on the room and like who’s there and what we’re discussing. But in general, I think races the top um on the foreground and then being a woman and then being queer (Merritt, Pos. 42).

This finding revealed participants’ responses to the questions: Can you please elaborate on how you rank your race, gender, and sexual orientation identities? Can you also provide a more detailed description of why you ranked it in this way? The concept of identity ranking became relevant when privileged people either perceived an individual’s Asianness, womanhood, or LGBTQness, or people discussed AAFSMs’ identity based on stereotypes and ignorance. Therefore, identity ranking was contextual, reflecting societal norms such as White-, heterosexual-, and masculine-supremacy. For instance, in a predominantly White campus, race comes first; in a larger, more diverse campus, gender might come first. Therefore, the more pressure an individual encounters, the more the individual seeks to express themselves. Whichever identity is most silenced and ignored tends to emerge as the most prominent.

### 5.3 Theme 3: reported experiences of constructed objectifications

Socially, as illustrated in the previous section, racial minorities were systematically disadvantaged and perceived as inferior due to race prejudice and/or ethnocentrism (Jones, 1997). Historically, Asian Americans experienced microaggressions through discriminatory slurs, such as yellow peril, model minority (Lee and Hong, 2020), forever/perpetual foreigners, honorary Whites (Tuan, 1998), and otherness/others (Wooden et al., 1983). Culturally, Asian Americans’ home cultures were seen as problematic (Lee, 2005). Living in a sociohistorical and sociocultural environment, AAFSM students were repeatedly objectified due to their race, gender, and sexual orientation. Although they share some degree of social experiences with women of color, LGBTQ women, LGBTQ of color, etc., Asian American female sexual minority students’ experiences with objectifications are distinctive.

#### 5.3.1 Subtheme 1: racially constructed objectification

Sociohistorically, Asian Americans have been perceived as the model minority. This racial stereotype, as one form of objectification, portrays all Asian Americans as super smart, contributing to the devaluation of AAFSM’s individuality. One participant explained,

In my case, the racism faced as someone who’s Asian, even now I was raised by white people. That doesn’t matter because I don’t look white. People are going to look at me and that’s a lot of what racism is. It’s on the surface like … they don’t see my personality. They don’t see who I am, what I do. All they see is my face and the fact that it doesn’t look like theirs. I really think it’s a matter of don’t judge the book by its cover, kind of, I don’t know if you’ve heard that phrase before. And in terms of Asians and especially the eastern Chinese people, it’s very much the idea of like a positive stereotype. They could like saying all Asians are good at math … it is still racist and it’s still bad. Because it’s a person making a generalization about an entire group of people that doesn’t necessarily apply to all of them. It takes away from our individuality as people, just because they’re saying something nice doesn’t mean it isn’t still bad (Lyric, Pos. 114).

Many participants have reported that they have confronted cultural rejection and marginalization. For instance, several participants shared that their cultures were pervasively perceived as exotic by White Americans although they were adopted and raised by a White family with American culture. Asian Americans were objectified (e.g., Asian Americans and their cultures are exotic) based on their “cover” regardless of whether they were adopted and raised by a White family or not. As Lyric indicated, racially constructed objectification occurs through White’s “generalization,” devaluation on Asian Americans’ “individuality,” and judgment “by its cover” (Lyric, Pos. 114). Another female bisexual Filipino American further explained, “The way I can explain it is the way I see racist people are very egocentric and just are too proud in their own culture and race” (Blake, Pos. 146).

One racially constructed objectification was captured in an interview with a Filipino American female bisexual master student who felt devalued due to “generalizations” made by White people. She narrated,

Um I think I mean no hate against like anyone who’s Chinese, Japanese, Korean, or any of those other things. But it made me feel invisible. Like it made me feel like the Philippines doesn’t really exist … So, it very much felt like I … our ethnicity doesn’t really exist. And it always made me feel like I was the same as everyone else, which isn’t bad (Merritt, Pos. 18).

All participants interviewed in this study reported that they were always objectified for merely being Asians regardless of other facets, such as family background. For instance, some participants preferred to be called American Asians, who had no connections with the Asian side, because they was adopted and raised in a White household; whereas some preferred to be perceived as Asians because they were highly bound with their home culture. Others preferred to be perceived as either Chinese, Korean, or Filipinos due to their distinctive experiences. Asian Americans are not a broad homogeneous group; instead, they have multiple subgroups.

Merritt along with other participants, further added that racial objectifications were also produced by White preferences. As Merritt continuously noted,

Like … Like I said, there’s no hate against like Chinese, Japanese, Korean folks. But we’re very different in terms of culture. And I think when people in particular assume that I’m Japanese or Korean, they wanna talk about J-pop or K-pop or K-dramas and things that are about like cultural element. And when they assume that I’m Chinese, they want to comment on like, the um political climate over there. Like, I’m not that I’m not actually that familiar. I don’t I’m not Chinese. Or they want me to speak Chinese with them because they speak Mandarin or something like, I don’t know any of this. Like, please leave me alone. I’m Filipino. I can barely even speak my own language. Um so I think when they ask me, in particular in my any of those, um was it like ethnicities? They want to connect with me because of those ethnicities (Merritt, Pos. 18).

Thus, participants reported that another manifestation of racial objectification stemmed from White preferences. In some cases, Asian American female sexual minorities were objectified as Chinese due to White preferences regarding politics or language; whereas, they were objectified as Korean or Japanese merely based on these preferences regarding cultures. People and society would be disappointed if they, as AAFSMs, did not fit into these objectifications. As Merritt said, “And when they found out I’m not, they get disappointed. Like it’s not my fault. You assumed. I’m sorry to disappoint you” (Merritt, Pos. 18). These mis-objectifications were reflective of racially constructed objectification experienced by study participants who were AAFSM in their educational and everyday lives.

Racially constructed objectification, as indicated above, conveyed messages to study participants who were AAFSM that Asianness was devalued, exotic, and marginalized. The examined racial-related objectification does not explicitly account for AAFSM objectified experiences because they also encountered sexual and gendered objectification.

#### 5.3.2 Subtheme 2: sexually constructed objectifications

Another theme emerged from the data analysis. Asian American women were stereotypically labeled as hyper-sexual. All participants indicated that they did not like this hyper-sexual label because “[n] obody fits into a specific box or specific label” (Charlie, Pos. 16). The feelings participants disclosed might be best characterized as sexually constructed objectifications—people and society mis-objectified Asian American women as only hyper-sexual. One Chinese American female sexual minority noted that the gendered and sexual objectification misrepresented who she was. The explanation was best captured in the following narrative offered by this participant,

I am demi-panromantic, demi-homosexual … So demi means demi as a prefix is under the asexual and aromatic umbrella. So that means that although I am panromantic, so my romantic spectrum is I’m pan romantic. So, I … I … I am attracted to everyone. It doesn’t matter the gender. It just matters like who they are as a person. Gender is not something that affects who I like. Versus like I believe like something like biromantic or bisexual is like gender or something that affects. It’s part of like what, you see as like what affects who likes and don’t like so. Me, it doesn’t and then homosexual wise like, I don’t like the penis, no penis. that’s just for me, a lot of people take it different ways. and then demi means that I, I do not usually like, have attraction. Like I’m not attracted to people generally. I have to get to know that person and have a connection with a person… like a full connection, like to know who they are and have spent time with them before I can develop an attraction to them, either romantically or sexually (Charlie, Pos. 10-16).

The study participants reported that they were objectified as hyper-sexual. However, some participants, such as Charlie, did not report that she was hyper-sexual, in terms of sexuality. Charlie indicated that she was demi-panromantic, needing to spend time with people to get to know each other before developing attraction. Sexual objectifications denied individuality and uniqueness, maintaining oppression on AAFSM students.

Furthermore, participants reported that fetishization was another form of sexually constructed objectification that they, as AAFSM, had to face. One participant noted,

Especially being an Asian female. I was fetishized a lot. I was, you know people would ask really outlandish questions about you know my private parts. And different things like that or they would say, I was like when I was dating, it was like I was a box that a lot of white men wanted to check off their list. Like oh I was with an Asian woman and I found a lot of men that wanted to be with me specifically because I was Asian that’s all they really cared about which to me was a huge red fly because I’m not a boxing check off your list … that’s not who I am. I’m a person with feelings and emotions and um so that was really … just a no go (Sherron, Pos. 14).

Sherron was sexually hyper-desirable, merely due to her womanhood and Asianness, by White Americans. This hyper-desirability of AAFSM facilitated the racial and gendered objectifications, which objectified them as commodities or checkboxes. This sexually constructed objectification also reinforced the objectification ideology held by White people, who believed that Asian American women were hyper-desirable because of the combination of womanhood and Asianness.

#### 5.3.3 Subtheme 3: gendered constructed objectification

In the male and female continuum, Asian American women and men were placed in two extreme endpoints. Asian American women were stereotypically viewed as hypersexualized, whereas Asian American men were perceived as undesirable. One participant indicated, “Asian men in America is like Asian men are like, not sexualized, right? Like they are … there is no sex appeal to Asian men. Asian women are hypersexualized” (Lennon, Pos. 40).

The same theme was echoed by another participant who studied at a predominantly White Midwestern university,

Asian women are attended to work kind of viewed as either like innocent versions or we’re seen as like nymphomaniac said there’s no in between. And like navigating that kind of landscape where we’re hypersexualized. But at the same time, we’re almost nonsexual to some people. But I think in particular its Asian men who are like non sexualized because of, I forgot what it was. But I think the historic context was white people were afraid of like Asian men taking white women. So, they … Asian men as like non masculine or like, um and nonsexual and whatever. And that kind of just spiraled on. So, it’s interesting to see that like Asian women are hypersexualized, but Asian men are not (Merritt, Pos. 30).

Gendered objectification is also socially constructed to perpetuate gender bias by promoting a sense of degrading. As Merritt elaborated, some Asian American women were objectified as hyper-sexual, whereas some were nonsexual. Asian American men were objectified as nonsexual because they were perceived as less masculine. All these aforementioned gendered objectification dehumanized Asian American females and males.

## 6 Discussion

The research findings show that Asian American female sexual minorities (AAFSM) disengaged from their Asianness, womanhood, and LGBTQ identities due to a lack of intersectional representations and their visibility from the mainstream community. Enhancing intersectional visibility and representation could foster a reconnection of AAFSM individuals with their own identities and break the binary world, which constrains the wholeness of the minority groups’ identities (Miville and Ferguson, 2006).

This paper utilizes an intersectional analysis to identity and understand the complex interplay of multiple dimensions of power that shape the experiences of AAFSM. This approach aligns with how power perpetuates entrenched discourses of privileged groups and reinforces dominant assumptions through various methods and strategies. These include applying pressure, threats, isolation, and segmentation to objectify marginalized identities, controlling curriculum contents and ideological orientations, and normalizing mainstream agendas through policies, norms, and discourses, all of which contribute to fostering subordination of the minorities (Royer and Chang, 2020).

The study reveals that participants experienced constructed objectifications, including gendered, racial, and sexual objectification. Their identities were not considered in isolation but were interconnected and influenced by multiple aspects of their social locations. The findings of this paper reflect the lack of intersectional representations in curricula, the contextualization of race, gender, and sexuality, and the reported experiences of constructed objectifications, which provide us a nuanced understanding of the participants’ experiences. The objectivations of the marginalized groups are operated through microaggressions in the form of using the discriminatory languages and discourse, such as yellow peril and model minority (Lee and Hong, 2020), denying Asian Americans as pure Americansm, devaluing the culture of the ethnic groups (Lee, 2005), and assigning labels to certain groups that do not accurately reflect their identities (Qin et al., 2008; Yeo et al., 2019).

The institutional practices, such as a lack of adequate physical protection and an openly hostile atmosphere toward sexual and racial minorities, have an impact on the self-perception and valuation of intersectional identities. The participants’ identities were devalued due to the absence of positive representation in university curricula (Jones, 1997; Yeskel, 1985).

The AAFSM’s experience of identity varied depending on the social settings they navigated, illustrating the dynamic nature of intersectional identities. Depending on the specific contexts, instances of labeling others as abnormal and devaluing homosexual orientations or minority racial groups can occur both unintentionally and intentionally to reinforce mainstream ideologies and social norms (Kosciw and Cullen, 2002; Kosciw, 2004; Kosciw and Diaz, 2006).

## 7 Conclusion

This study provides a profound exploration into the intricate experiences of the unmarked Asian American female sexual minorities (AAFSM) at Midwestern universities in the United States, emphasizing that their distinctive voices that should be heard. The intersectional analysis in this study provides a comprehensive understanding of the multi-dimensional experiences of AAFSM students. Through the application of an intersectional lens, this study uncovers the multifaceted ways in which race, gender, and sexual orientation intersect to shape the identities and educational experiences of the AAFSM students within various social contexts.

The findings of this study illustrate the pervasive nature of constructed objectifications, including gendered, racial, and sexual dimensions, which the participants face. They highlight the contextualization of participants’ identities based on situational factors. They reveal the devaluation of the AAFSM students’ Asianness, womanhood, and LGBTQness due to the absence of positive representation in the curriculum.

## 8 Implications

The findings provide crucial information for stakeholders and policymakers to develop intersectional interventions that foster an inclusive educational environment. To achieve this goal, universities may consider the following suggestions: Firstly, universities should develop intersectional curricula that are culturally responsive and inclusive. This entails creating learning materials that authentically reflect a diverse range of identities and their often-overlooked experiences. Secondly, universities should amplify the visibility of underrepresented groups within course materials, readings, and their narratives, highlighting their significant contributions and perspectives.

Curricula should integrate intersectionality to make visible the complexities of intersecting identities and enhance representation across race, gender, and sexuality. For instance, many schools currently lack LGBTQ-inclusive content for sexual minority students. Similarly, Asian American history and the histories of other ethnic groups are often omitted from American history curricula. While some schools have begun to include LGBTQ perspectives, women’s studies, or Asian American history within broader historical frameworks, this study considers these steps as incremental improvements. The study suggests that enhancing intersectional representation across race, gender, and sexuality could facilitate a more holistic awareness of these identities among students and educators alike.

Participants indicated that being the minority groups in the predominantly White universities posed significant challenges as they navigated the intersections of racism, sexism, and heterosexism. Universities should educate individuals to be aware of new cultures and knowledge of the minority groups, and to break the societal stereotypes and biases that are deeply rooted in various locations in universities. Universities should offer comprehensive training and support services to enhance faculty and staff awareness of the distinctive challenges faced by underrepresented groups. Additionally, universities should establish specialized support services, including counseling, mentorship programs, and safe spaces designed specifically to support marginalized students.

It is important for universities to regularly conduct campus climate assessments to identify any issues or challenges that may impact marginalized students and to take proactive steps to address these concerns. Additionally, universities should review their policies to remove any discriminatory elements and biases.

It is essential for universities to promote the involvement of the marginalized students in university governance and decision-making processes. It ensures that their perspectives are considered and integrated into university policies, curriculum development, and campus culture. By encouraging marginalized students to actively participate in shaping campus life, universities can ensure that their voices are heard, and their contributions valued.

## 9 Significance of the study

The study provides discussions that contribute to creating an inclusive campus environment for AAFSMs. It offers meaningful suggestions for educators, administrators, policymakers, and stakeholders to foster an equal and equitable educational environment and to address the multifaceted challenges faced by AAFSM students.

The study contributes to the literature by providing an in-depth exploration of the unique experiences of AAFSM students, a group that has been largely overlooked in existing research. It advances the understanding of how intersectionality can be applied to study and address the educational experiences of marginalized groups. Further, it contributed to advancing research on intersectionality by placing emphasis on AAFSM college students in Midwestern universities in the United States.

The study also established a new field of scholarship dedicated to the study of AAFSM, thus enriching intersectionality scholarship. Its significant contributions include the recognition of the absence of intersectional representations in curricula, identification of constructed objectifications, and contextualization of race, gender, and sexual orientation.

## Data Availability

The raw data supporting the conclusions of this article will be made available by the authors, without undue reservation.
